# Smaller, Scale-Free Gene Networks Increase Quantitative Trait Heritability and Result in Faster Population Recovery

**DOI:** 10.1371/journal.pone.0014645

**Published:** 2011-02-09

**Authors:** Jacob W. Malcom

**Affiliations:** Integrative Biology, University of Texas at Austin, Austin, Texas, United States of America; University of Utah, United States of America

## Abstract

One of the goals of biology is to bridge levels of organization. Recent technological advances are enabling us to span from genetic sequence to traits, and then from traits to ecological dynamics. The quantitative genetics parameter heritability describes how quickly a trait can evolve, and in turn describes how quickly a population can recover from an environmental change. Here I propose that we can link the details of the genetic architecture of a quantitative trait—i.e., the number of underlying genes and their relationships in a network—to population recovery rates by way of heritability. I test this hypothesis using a set of agent-based models in which individuals possess one of two network topologies or a linear genotype-phenotype map, 16–256 genes underlying the trait, and a variety of mutation and recombination rates and degrees of environmental change. I find that the network architectures introduce extensive directional epistasis that systematically hides and reveals additive genetic variance and affects heritability: network size, topology, and recombination explain 81% of the variance in average heritability in a stable environment. Network size and topology, the width of the fitness function, pre-change additive variance, and certain interactions account for ∼75% of the variance in population recovery times after a sudden environmental change. These results suggest that not only the amount of additive variance, but importantly the number of loci across which it is distributed, is important in regulating the rate at which a trait can evolve and populations can recover. Taken in conjunction with previous research focused on differences in degree of network connectivity, these results provide a set of theoretical expectations and testable hypotheses for biologists working to span levels of organization from the genotype to the phenotype, and from the phenotype to the environment.

## Introduction

A primary goal of biology is to bridge levels of organization. Biologists working in the arena of cell and molecular biology tend to focus on the genotype-phenotype map (GPM), whereas ecologists tend to focus on the phenotype-environment map. The phenotype is the common interface between these two mappings. We would like, ultimately, to have an understanding that spans from genotypes to the environment, including an understanding of how the environment (which encompasses both biotic and abiotic components) impacts genotypes; focusing on the causes and consequences of phenotypic evolution is a prime starting point. The presence of heritable variation in organismal phenotypes is a key component of evolution [1]–[3]. The degree to which a trait is heritable plays an important role in predicting the response to selection: the higher the heritability, the faster a trait can change, whereas lower heritabilities slow phenotypic change and minimize the effects of external perturbations [2], [4], [5]. Knowing a trait's heritability is important to many fields, such as agricultural sciences—in which artificial selection is used to maximize a desirable trait—and evolutionary ecology, in which we want to understand how evolution and ecology interact to drive patterns seen in nature [6]–[8]. The ratio of genetic (additive or total) variance to phenotypic variance describes the (narrow- or broad-sense) heritability of a trait. It is a mathematical fact that a given level of genetic variance may be achieved with any number of loci [1]. A different question is whether or not this actually occurs, and if there are any further implications of dividing the variance across a variable number of loci.

Assume that two species each possess an ecologically-analogous trait, but that due to their different evolutionary histories, the number of genes underlying variation in the trait is twice as large in one species as it is in the other. The first implication of this assumption is that the average contribution of each gene to trait variation is smaller in the species with more underlying genes. We know that the rate of change of genetic variance is inversely proportional to the number of underlying loci [9], [10], and therefore the species with twice as many loci will lose gene variation at approximately half the speed of the other species. If the GPM is purely linear, then heritability should be minimally affected because the proportion of phenotypic variance lost (due to genetic variance lost) will be the same.

**Figure 1:**
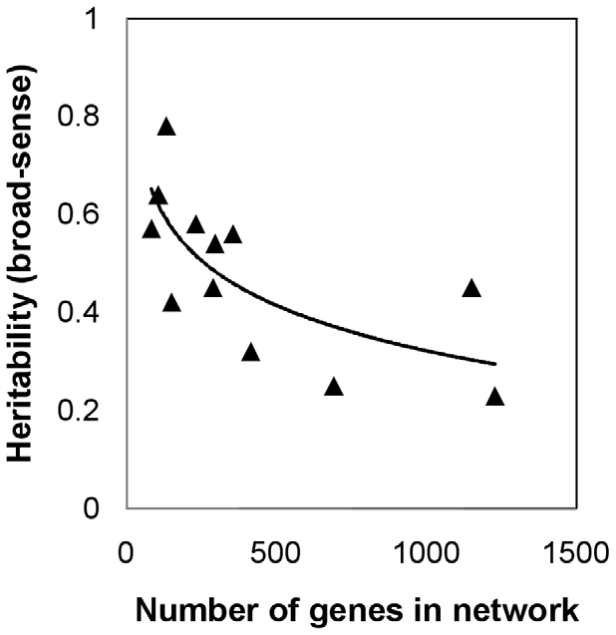
Fruitfly trait heritability as a function of network size. The broad-sense heritability for twelve quantitative traits in *Drosophila melanogaster* as a function of the estimated size of the underlying gene regulatory network. When network topology (average connectivity) is considered in a regression model, R^2^≈0.68 (*p* = 0.023). Note that the data points are drawn from what must be considered an exploratory, hypothesis-generating method that requires extensive testing to confirm the networks. Data from [16]–[18].

We have reason to believe that the GPM is not purely linear, however, but is better-represented as a hierarchical network of nodes (genes) and edges (functional relationships) [11]–[13]. That is, rather than viewing the GPM as a list of genes with arrows pointing directly from each gene to a trait, some genes have arrows pointing to other genes, which may point directly to the trait, or may point to other intermediary genes before the path arrives at the trait. This has been termed a multilinear model [14], [15], such that the GPM is not nonlinear per se, but is more complex than a purely linear model as described above. If these networks introduce directional epistasis, which “hides” and “releases” genetic variation [14], [15], then the effect of a change in genetic variance may not have a 1∶1 impact on phenotypic variance (specifically, the portion of phenotypic variance attributable to genetic variance). Gene regulatory networks are thought to follow a scale-free (i.e., power law) degree distribution [11]; we can hypothesize that variation in network topology alters path lengths across the network and therefore systematically affects the degree of epistasis. Together, these underlying principles suggest that the number of genes and their relationships in a network could both affect heritability. Data from the Mackay lab for a dozen *Drosophila melanogaster* traits [16]–[18] suggests that a relationship between network characteristics and heritability exists (Figure 1).

Ecologists increasingly recognize that evolution can alter ecological dynamics (and vice-versa), rather than considering distinct ecological and evolutionary time-frames [19]–[22]. Gomulkiewicz and Holt [23] formalized the link between trait heritability and population adaptation after an environmental change, showing that higher heritabilities, which confer more rapid adaptation, decrease the chance of extinction due to maladaptation. The basic model has been extended to include cases of phenotypic plasticity [24], as well as the implications of differing heritabilities for community assembly [22], [25]. Bell and Gonzalez [26] recently tested the general hypothesis of population rescue by evolution using yeast and found the U-shaped curve of population decline and recovery predicted by Gomulkiewicz and Holt.

### Previous research and current goals

Prior research has considered gene networks underlying quantitative traits, rather than purely linear genotypes. Here I describe three papers that are most-similar to the questions I consider. Each of these is different from the present contribution in that the authors focused on variation in the density of connections among the genes in the networks rather than variation in the number of genes or the overall degree distribution. Frank [27] examined the evolution of networks that are selected based on their ability to produce different phenotypes in two distinct life periods. Unlike the present model, which considers only one input per gene but multiple outputs, Frank's analysis was primarily concerned with the evolution of the number of inputs per gene. He found that trait heritability was a function of an interaction between the average number of inputs per gene and mutation rate, with maximum heritabilities at intermediate mutation rates. Although he tested several network sizes, he did not find an effect on developmental evolution and did not test for an effect of network size on heritability. Kimbrell and Holt [28] used a model similar to that of Frank, but considered the ecological implications given a source patch in which individuals are adapted to the environment and a novel patch which functions as a sink (negative population growth) until an adapted population has evolved. They found that network connectivity among 10 genes could drastically alter the ability of individuals canalized to the source patch to adapt to the novel conditions of the sink patch; specifically, lower connectivity conferred a higher rate of adaptation than high connectivity. Most-recently, Repsilber and colleagues [29] considered a trait underlain by networks of 3–10 genes and varying connectivity. Among their conclusions was the fact that network architecture was related to rates and accuracy of adaptation, as well as habitat heterogeneity. They did not examine the link between network size and trait heritability, and although they varied network size, the computational complexity of their model limited the maximum size to ten genes, far smaller than the size of currently estimated networks for organismal phenotypes.

This contribution is focused on the effects of two network characteristics, size and topology (distribution of the out-degree of genes), on heritability and population recovery times. There are two basic goals: first, I examine the characteristics of a network model of genetic architecture as compared to purely linear genotypes (as might represent classical quantitative and population genetics). Second, I test two central hypotheses: 1., The heritability of a quantitative trait is primarily determined by the size and topology of the underlying gene network when the population exists in a stable environment; and 2., Specifying the network underlying a quantitative trait, rather than heritability, produces population recovery patterns similar to that of previous research in which heritability is specified. I test the hypotheses using an agent-based model in which individuals in a single population possess an ecologically-important trait that is encoded by a Boolean network of varying topologies, and sizes ranging from 16 to 256 genes. In order to maintain computational tractability I simplify network connectivity such that any gene is regulated by a single upstream gene and feedback is not present. This latter characteristic is the same as the estimation of Directed Acyclic Graphs, a common bioinformatics approach in systems biology [30]. First, the results support the analytical solutions relating number of underlying loci to the rate of change of genetic variance, but the rates of change are lower, relative to linear genotypes, with the directional epistasis that network structures introduce. Second, I recover a negative relationship between network size and heritability. Third, this network-heritability relationship scales up to population recovery after an environmental change, such that species with small networks have higher heritability and faster recovery. The extension to ecological outcomes suggests that integrating the information that genomic methods afford (e.g., identifying the genes underlying a quantitative trait) can expand our understanding of evolutionary ecological dynamics in a mechanistic manner.

## Results

### Basic genetic architecture performance

**Figure 2:**
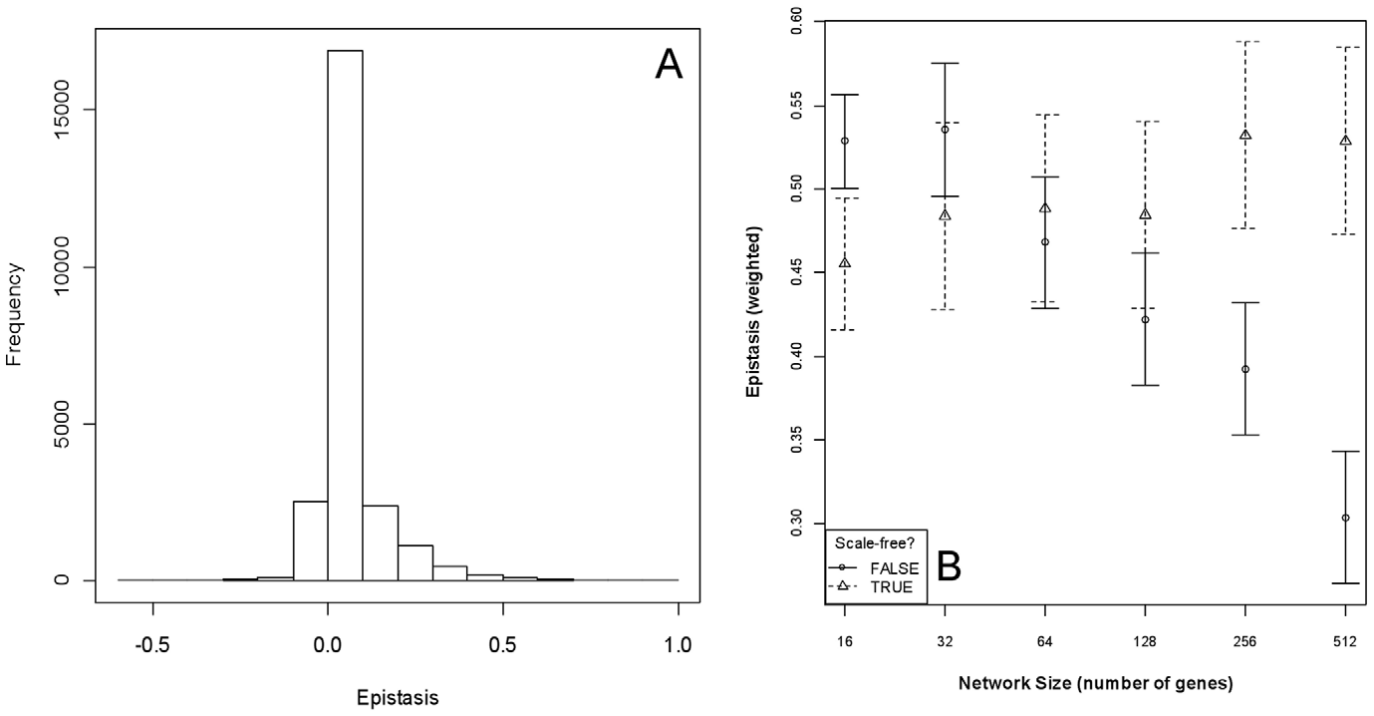
Epistasis in the Boolean gene networks used in these models. Panel A shows the distribution of 24000 epistasis estimates across all network sizes and topologies. Directional epistasis is prevalent (94% of all single- versus double-mutants). Panel B shows mean weighted epistasis (±95% CI) as a function of network size and topology. The weighting, with weights calculated as the standard deviation of epistasis within network size, was required to achieve homoskedasticity for statistical analysis. Without weighting, a strong negative relationship between epistasis and network size is observed (data not shown).

Negative epistasis was observed (5% of cases), but positive epistasis was far more common (89% of cases) in these networks (Figure 2A). The amount of epistasis was strongly related to the size and the topology of the network and an interaction of the terms. Weighted epistasis increases slightly with network size when the networks possess a scale-free out-degree distribution, but declines with network size when network topology is random (Figure 2B). (Interestingly, if epistasis is not weighted—the weighting is required to meet the assumption of homoskedasticity for the preceding analysis—then epistasis declines with network size for both scale-free and random networks.) Epistasis is absent if we assume the GPM is purely linear.

**Figure 3:**
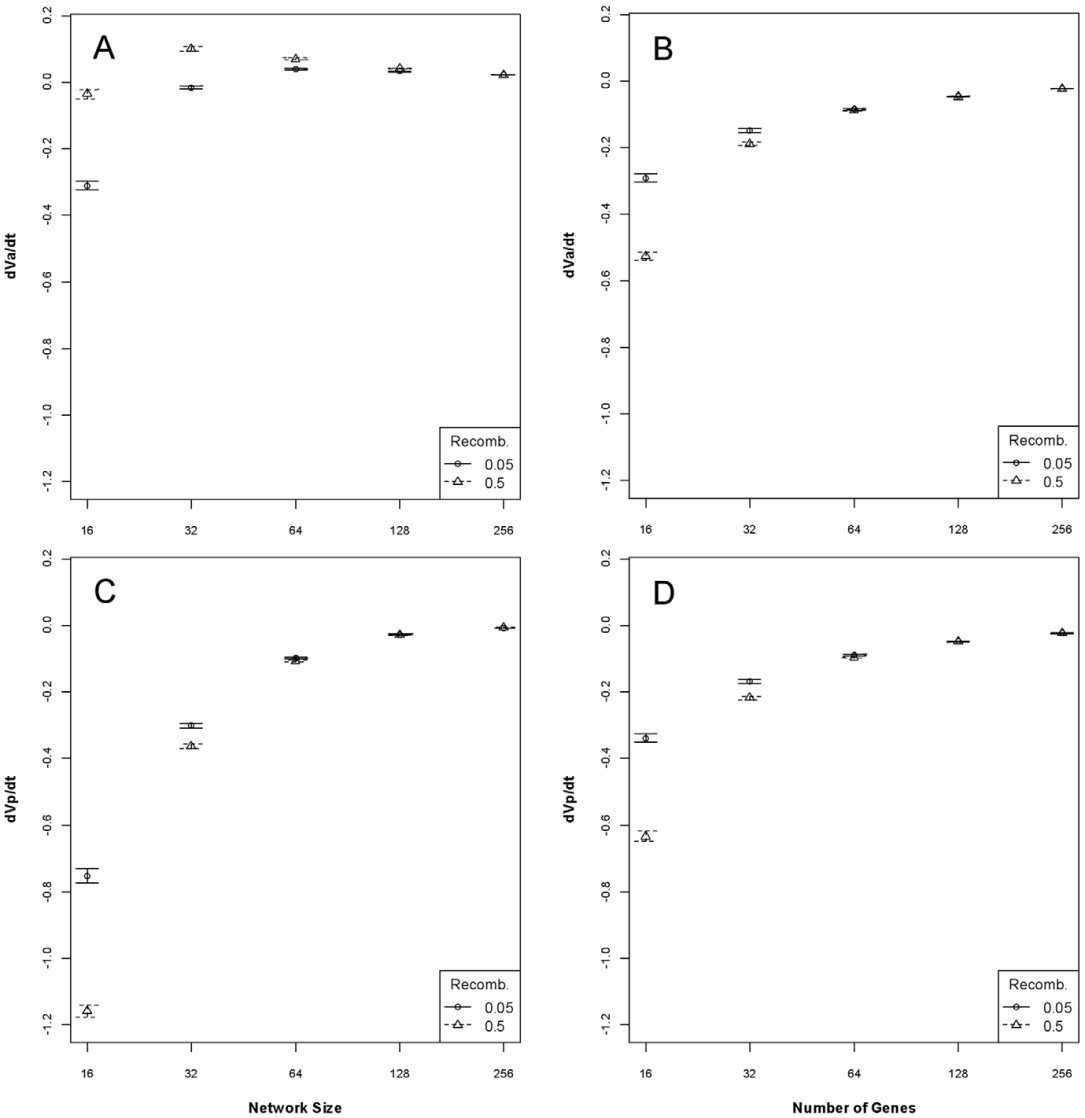
The rates of change of genotypic and phenotypic variance over 250 generations in a constant environment. On the left, the mean (±95% CI) rate of change of additive genetic variance (dV_A_/dt; Panel A) and phenotypic variance (dV_P_/dt; Panel C), given a network genetic architecture, as a function of network size and recombination rate. On the right (Panels B and D), the same parameters given a linear genetic architecture. The results are generally consistent with the analytical solutions assuming additivity of Crow and Kimura [9] and Bürger [10].

The rate of change of additive genetic variance during the first 250 generations of the simulation was strongly predicted from the number of underlying genes given both network and purely linear genetic architectures (R^2^ = 0.81 [*P*<2.2e^−16^] and R^2^ = 0.95 [*P*<2.2e^−16^], respectively). Topology (random vs. scale-free) is important when the genetic architecture is a network (Figure 3A) and the width of the fitness function is a minor factor. This is in contrast to linear genetic architecture where there is no topology and fitness function width is an important factor (Figure 3C). Notably, the rate of change of additive genetic variance tends to be much lower (approximately half) given a network GPM relative to a linear GPM. In addition, for these simulations, additive genetic variance actually increased (to varying degrees) in 32-gene and larger networks. In contrast, the phenotypic variance rate of change is approximately twice as fast given a network GPM relative to a linear GPM across all but the largest networks (Figures 3B and 3D).

More important with respect to the topic of quantitative trait heritability is the comparison of the genetic and phenotypic rates of change within a category of GPMs. Given a linear GPM, the rates of change of additive genetic variance and phenotypic variance are very similar (Figures 3C and 3D); as a result, heritabilities are only slightly different between network sizes (see next section). By contrast, the phenotypic variance rate of change is much faster than the additive genetic variance rate of change given a network GPM at small network sizes and the rates only begin to converge as network size becomes large (Figures 3A and 3B). As such, substantial additive genetic variance remains hidden by the network topology even when phenotypic variance is rapidly removed.

### Focal hypotheses

#### Hypothesis 1

**Figure 4:**
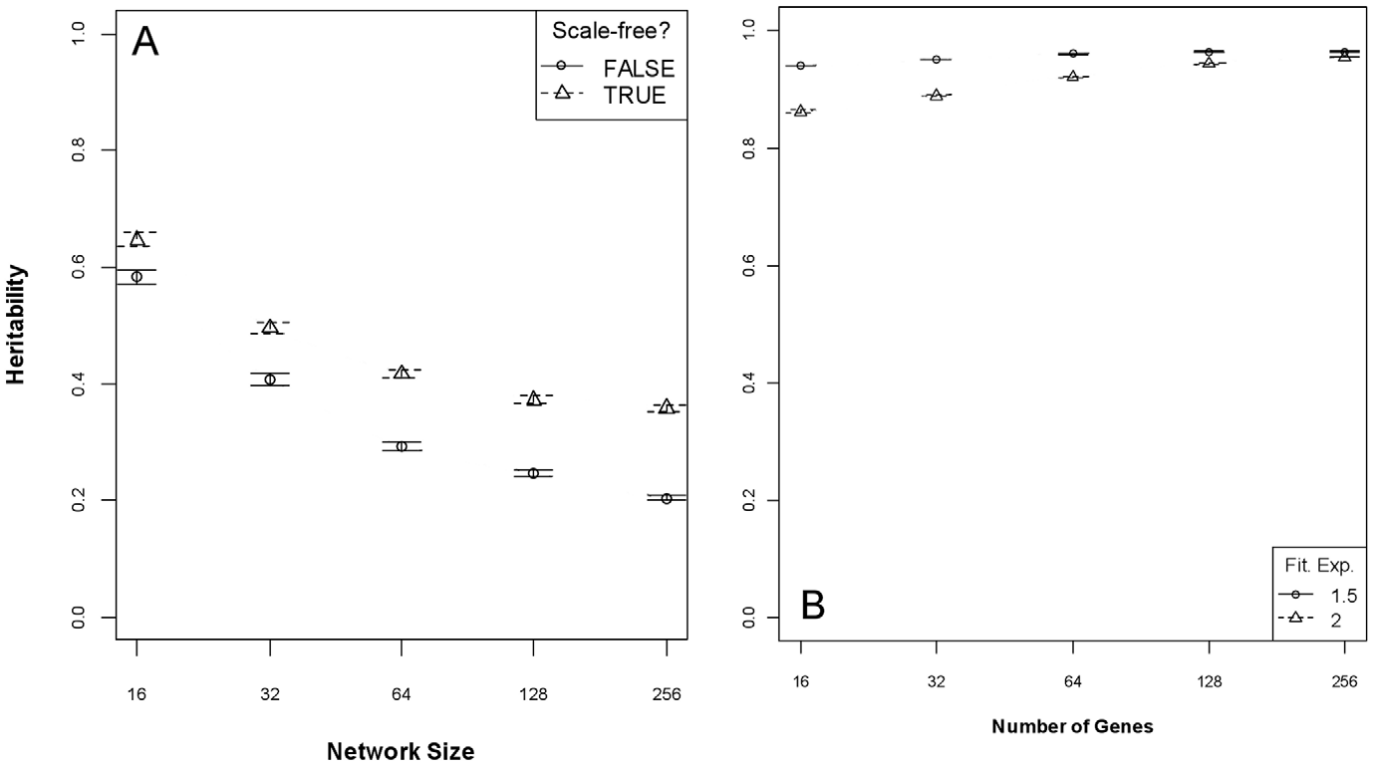
Quantitative trait heritability after 250 generations, given network (A) or linear (B) genetic architectures. The mean heritability (±95% CI) of the ecologically-critical trait when the genetic architecture is defined as a network, as a function of network size and topology; smaller networks and scale-free topology increase heritability (Panel A). The mean heritability (±95% CI) of the trait when the genetic architecture is purely linear, as a function of number of genes and the width of the fitness function (see Methods). Contrary to the network architecture, when the architecture is purely linear heritability is (weakly) positively related to the number of underlying genes.

Differences in the initial levels of additive and phenotypic variance, and differences in the rate of change of each variance component, results in systematic differences in the average trait heritability in a constant environment. The number of genes in the network, the topology of the network, the recombination rate, and average additive genetic variance before the environmental change account for 83% of the variance in average heritability of the quantitative trait in the 50 generations before the environmental change (Table 1). Smaller networks, scale-free network topology, and low recombination rate all confer higher heritability (Figure 4A). The term for additive genetic variance was not significant on its own (*p* = 0.41), but an interaction with network size was significant and accounted for 7% of the variance in heritability. Note that the global model (all predictors and first-order interactions) possessed the lowest AIC by 300 points, but with 240 model terms, was far less interpretable than the AIC next-best model with network size, network topology, recombination rate, average additive variance, and interactions as predictors.

**Table 1:** Factors influencing quantitative trait heritability in a stable environment.

Factor	Effect	% Variance	*p*-value
	Direction	Explained	
Network Size	(−)	47	<2.2 e^−16^
Network Topology	(−)	10	<2.2 e^−16^
Recombination Rate	(−)	14	<2.2 e^−16^
Pre-change V_A_	( )	0	0.41
Network Size * V_A_	(+)	7	<2.2 e^−16^

*Effect direction* refers to whether the relationship between heritability and the predictor is directly or inversely proportional. Additional interaction terms not presented here accounted for the remaining 5% of variance, but each at <1%.

In contrast, heritability increases with an increasing number of genes in simulations where genetic architecture is linear, but the rate of change of heritability relative to the number of underlying genes is small (about ¼) relative to the rate of change given a network GPM. Parameter estimates for linear versus network number of genes and heritability were 2.1e^−4^ and −9.1e^−4^, respectively. (Note that in order to arrive at these parameter estimates, I treated network size as a continuous variable, as opposed to treating it as a factor in all other analyses; see *Methods*.) In addition, the width of the fitness function plays a role in determining heritability under a linear genetic architecture but not a network (Figure 4B).

#### Hypothesis 2

**Figure 5:**
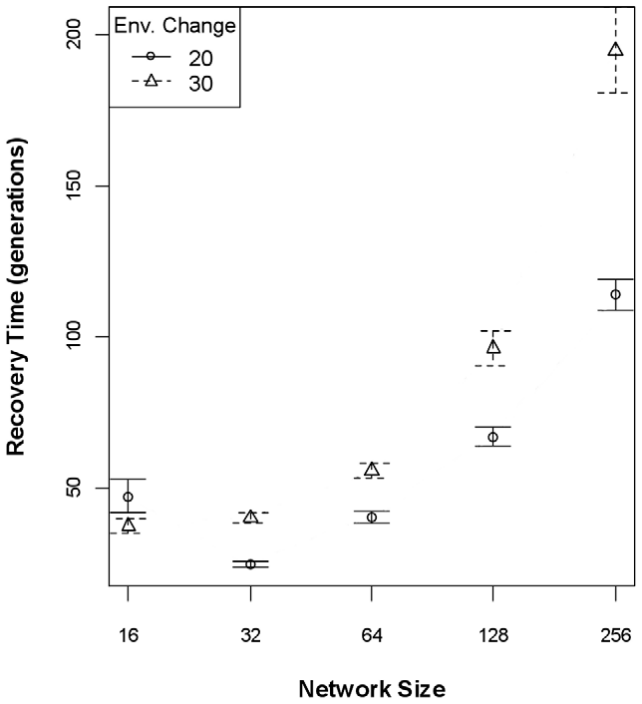
Population recovery times given network genetic architectures. Mean time (±95% CI) required for a population to recover to pre-impact population size after a sudden environmental change when the genetic architecture is defined as a network, as a function of network size and the degree of environmental change (dE; arbitrary units). Population recovery takes long if either network size or the degree of environmental change is greater.

Population recovery times following a sudden environmental change are primarily determined by network size, the degree of environmental change, the average additive variance and an interaction between network size and the width of the fitness function (Table 2). The full-interaction model R^2^ = 0.90 (*P*<2.2 e^−16^), but most individual terms explained little variance alone; Table 2 is based on a simpler model with much lower AIC (two hundred points lower) including only network size, fitness function, average pre-change additive genetic variance, and degree of environmental change (R^2^ = 0.74, *P*<2.2e^−16^). Populations in which the underlying network is small generally recover from the sudden environmental change fastest (Figure 5); recovery takes longer the more severe the environmental change, and recovery is faster when the fitness function is wider. The notable exception is the higher average recovery time for the 16-gene networks, which is a result of some populations going extinct after the change in some simulation runs (i.e., they never recover). In these cases, it appears that the rate of change of mutational variance is much lower than the rate of change of genetic variance given the small networks, all variation is lost during canalization, and without variation the trait cannot evolve. When populations survive the environmental change, however, there is a surge in additive variance after the environmental change. The degree of environmental change enters the model for predicting heritability (larger impact = larger change in heritability), but the proportion of variance explained is very small and the overall model fit declines (R^2^ = 0.772; Table 3). When the genetic architecture is linear, population recovery after a sudden environmental impact is nearly identical to the scenarios in which genetic architecture is represented as a network.

**Table 2:** Primary factors influencing population recovery time following a sudden environmental impact.

Factor	Effect	% Variance	*p*-value
	Direction	Explained	
Network Size	(+)	25	<2.2 e^−16^
Fitness Function Width	(−)	11	<2.2 e^−16^
Pre-change V_A_	(+)	10	<2.2 e^−16^
Environ. Change	(−)	1	1 e^−4^
Env. Cange × Fit. Width	(−)	12	<2.2 e^−16^

*Effect direction* refers to whether the relationship between population recovery time and the predictor variable (Factor) is directly or inversely proportional.

**Table 3:** Factors influencing quantitative trait heritability following a sudden environmental impact.

Factor	Effect	% Variance	*p*-value
	Direction	Explained	
Network Size	(−)	47	<2.2 e^−16^
Network Topology	(−)	13	<2.2 e^−16^
Recombination Rate	(−)	17	<2.2 e^−16^
Environ. Change	(+)	1	2.7 e^−6^

*Effect direction* refers to whether the relationship between heritability and the predictor is directly or inversely proportional.

**Figure 6:**
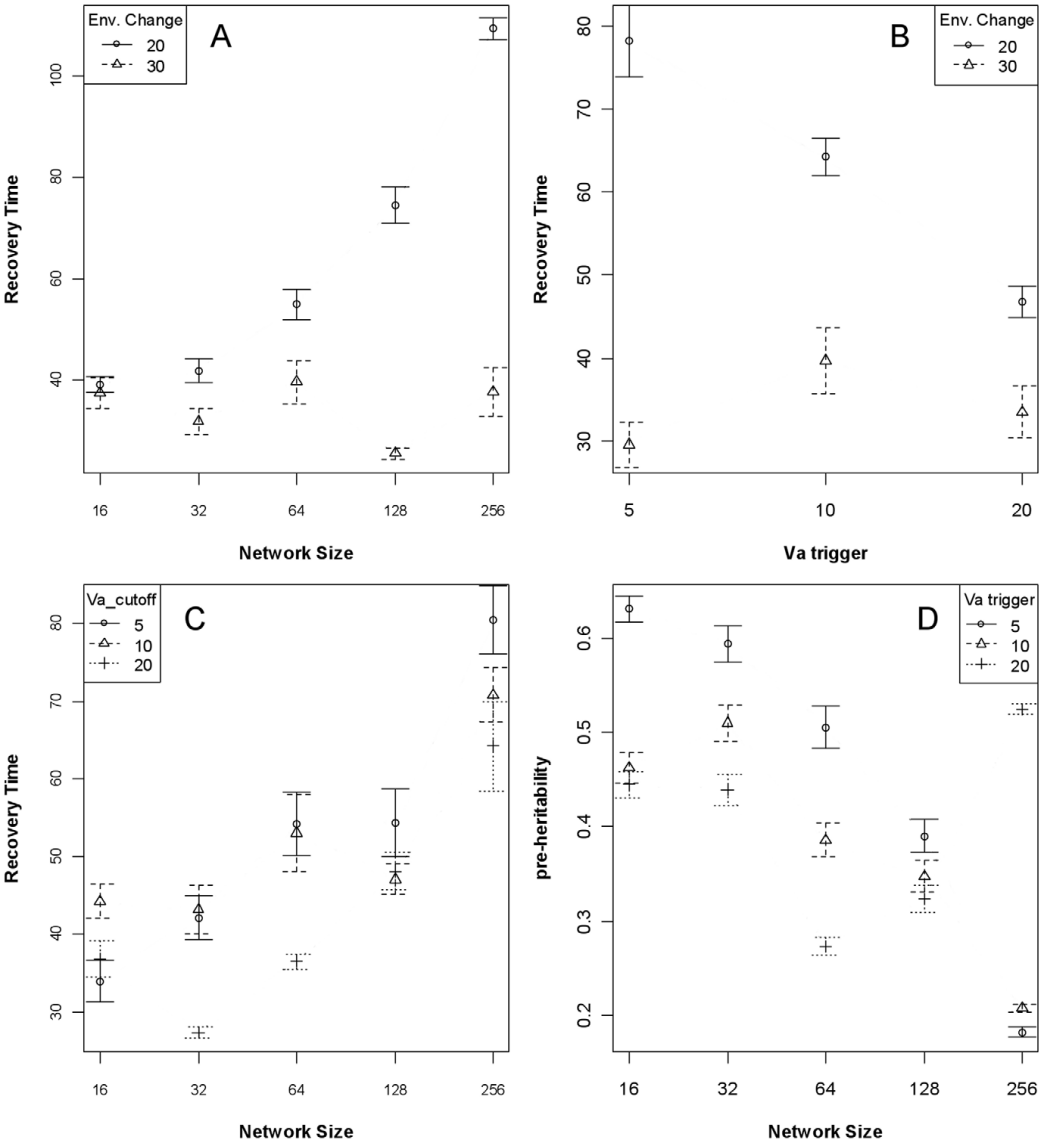
Recovery time (A-C) and pre-change heritability (D) when experimentally controlling for additive genetic variation. When the environmental change is smaller (20 units), the relationship between network size and recovery time is clear, but when the impact is larger (30 units), the relationship is lost (Panel A). Panel B partitions recovery time between the additive genetic variance trigger (V_A_ trigger) for the environmental change and degree of environmental change, demonstrating the effect of the environment with respect to the role of V_A_. Averaging over degrees of environmental change and considering recovery time as a function of network size and V_A_, the positive relationship between network size and recovery time is more apparent that solely considering the V_A_ (Panel C). Panel D shows that, although there is an equivalent amount of additive variation present in the population for each network size, smaller networks tend to have higher pre-change heritability (i.e., pre-heritability) than larger networks.

Given that the rates of change of additive genetic variance is dependent on network size (and topology), a natural objection is that there are different levels of additive variance at the time of environmental change (i.e., at 250 generations). This is true: smaller networks possess more additive variance than larger networks, and the amount of additive variance explains a small portion of recovery time. In addition to the statistical control introduced by including the pre-change additive genetic variance in the models discussed above, to experimentally control for the differences, I ran another set of simulations in which the environment changed when the population's V_A_ crossed thresholds of 5, 10, or 20. The environmental change did not occur by the 1500^th^ generation for some runs because the V_A_ had not reached the threshold, and those simulations were terminated. A large number of simulations did work for all network sizes, however: for network sizes 16, 32, 64, 128, and 256 there were, respectively, 216, 187, 193, 214, and 178 successful simulations. Two interesting results were apparent. First, the degree of environmental change plays the largest role in determining recovery times (Table 4), and essentially de-couples the relationship between either network size or pre-change additive genetic variance and recovery time when the impact is large (Figure 6a, b). If we average over the different degrees of environmental change, then the relationship between network size and recovery times is more apparent than the effect of different levels of additive genetic variance (Figure 6c), as is born-out in Table 4. Second, pre-change heritability tends to be higher for smaller network sizes, generally without respect to V_A_ threshold (Figure 6d). The exceptionally-high value for 256-gene networks with a V_A_ threshold of 20 likely stems from the small number of successful simulations with this combination (*n* = 34, whereas most other combinations *n* = 72).

**Table 4:** Factors influencing recovery time when the environment changes at a given (5, 10, or 20 units) level of additive genetic variance.

Factor	Effect	% Variance	*p*-value
	Direction	Explained	
Network Size	(+)	4	5.5 e^−15^
Pre-change V_A_	(−)	2	6.0 e^−11^
Environ. Change	(+)	24	<2.2 e^−16^
Net. Size * Env. Change	( )	12	<2.2 e^−16^
Pre-V_A_ * Env. Change	( )	6	<2.2 e^−16^

*Effect direction* refers to whether the relationship between heritability and the predictor is directly or inversely proportional.

## Discussion

Biology is rapidly approaching the stage at which data can be gathered from the level of entire genome sequences up through communities [31]–[33]. Successful integration will require bridging at least three distinct levels of organization, the genotype, the phenotype, and the ecotype (i.e., the environment). I suggest here that heritability could be a bridging concept, and test the hypothesis using a series of agent-based models, comparing trait heritability of network-based genetic architecture against scenarios in which the genotype is purely linear (additive), and examining the end effect on population recovery after a sudden environmental change. The results suggest that variation in the number of genes and the network topology can drive differences in population recovery rates, for two reasons. First, as variation is partitioned among a greater number of genes, the average effect of each gene is reduced and the rate of phenotypic change is reduced. Second, the directional epistasis introduced by the network architecture distorts the GPM so that additive genetic variation evolves differently—i.e., is hidden and released—than how phenotypic variation evolves. These two factors conspire to affect the trait's heritability, which, as a measure of the potential rate of phenotypic change, describes how quickly populations should recover.

The extensive directional epistasis observed with these networks parallels the empirical and computational results observed by He and colleagues [34]. Also consistent with numerical analysis [35], epistasis declines dramatically with increasing network size: the smaller the mutational effects—as a result of the larger network dividing the task among more genes—the greater the reductions in epistatic effects. As Carter and colleagues encouraged based on their analysis [14], empirical research should consider that directional epistatic variance can be converted to additive variance and thereby influence offspring phenotypes.

Regardless of the specification of genetic architecture class—linear versus network—the rate of change of genetic variance is inversely related to the number of genes underlying the trait, in agreement with the analytical results of Crow and Kimura [9] and Bürger [10]. There were, however, significant differences in the rates of change between purely linear and network genetic architectures, most strikingly in that the rate of change of genetic variance in networks was about one-half the rate of change in linear architectures. In contrast, the rate of change of phenotypic variance was nearly twice as fast given a network as it was given a linear genotype. This highlights the conjecture that networks inherently introduce redundancy [36] and the analytical conclusion that epistasis hides (and reveals) variation [14], [15], [37]. The network architecture permits a more rapid approach to an optimal phenotype while maintaining more genetic variability.

### Heritability

The effects of directional epistasis hiding and releasing additive genetic variation, and doing so increasingly more as network size increases, results in heritability being regulated by network size and, to a lesser degree, topology and recombination rate. This holds true whether heritability is measured after a 250-generation canalization period or an arbitrary period of time before the population reaches a given level of additive genetic variance. Does the number of underlying genes influence heritability in real organisms? Importantly, the observed negative relationship between network size and heritability is the same direction as for *Drosophila* data (Figure 1), but examples of the same traits from different taxa are needed and should become available in the future. These results, although based on a simplified network model (see Caveats and Conclusion), suggest an empirically testable hypothesis: if no correlation exists, then classical, purely additive models are most appropriate, but if a negative correlation is still apparent, then networks offer a better model. Observing such a correlation does not guarantee that the model used here is the generating model of reality—and it likely is not truly correct, due to the simplified network structure—but should be indicative of considering network size (and connectivity) into the future.

The apparent relationship between network size and heritability suggests that “evolvability” should be evolvable by the processes of gene duplication and gene loss [38]–[41]. Heritability evolves as genetic variance increases or decreases with respect to a given level of phenotypic variance, and the rate of change due to these changes should be faster than the (typically) much slower rate of gene duplication and loss. But possible rates of change, and the range of heritabilities that can be explored in a given time-frame, should be regulated in part by the number of genes underlying a trait. Species vary widely in genome size, the number of estimated genes (e.g., [42]), and the number of alternative isoforms from splicing [43]. This suggests that there could be variation in the size of networks underlying particular traits; selection for higher trait heritability should favor individuals whose networks are smaller. A test of this hypothesis may consist of estimating the gene regulatory networks underlying one or more traits in several “closely” related species, e.g., the sequenced *Drosophila* [42], and testing for both differences in network size underlying particular traits and a negative relationship between network size and heritability. Kellermann and colleagues [44] recently showed correlations between range size and the heritability of cold and desiccation resistance traits in *Drosophila*; we can hypothesize that some proportion of those differences are rooted in the sizes (and topology, connectivity) of the underlying gene regulatory networks for the traits.

### Population Recovery

The rate of adaptation to novel ecological conditions—as modeled here—may be an important factor in the face of sudden landscape modifications or, more generally, with respect to source-sink dynamics in a multi-patch environment [28], [45], [46]. In general, the smaller the network, the higher the heritability, and, as expected [23], the faster the population recovery from a sudden environmental change. But the role of heritability, and more specifically the additive genetic variance present in the population, tends to be less of a factor than the number of underlying genes, which regulates the rate of change of genetic variance [9], [10]. There must be additive genetic variance present in the population for any evolution to occur, but whether that variance is distributed across a few or many genes is important to the rate of trait evolution and population recovery because the average contribution of each gene is inversely proportional to the size of the network in which it is a player. The increase of additive genetic variance after the environmental change (which induces a population bottleneck) keeps with the analytical results of Naciri-Graven and Goudet [37] and several experimental studies that they reference. A straight-forward empirical and experimental test of this simulation result—if, in fact, network size underlying a particular trait varies between species—is to find two species with different-size networks, equalize additive variance for the trait, and then measure the response to selection. If responses are not identical, and specifically if the response is slower for the species whose underlying network is larger, then the hypothesis of the importance of network size is supported. The general results also hold for network connectivity, a scenario which has been examined in greater detail by Kimbrell and Holt [28] and Repsilber and colleagues [29]. These simulation results may be tested by comparing the recovery times of two similar species with different size (and different topology) networks for a trait, e.g., examining a second yeast species in an experiment paralleling that of Bell and Gonzalez [26].

### Caveats and conclusion

Like all models, these simulations are simplified versions of reality, and these simplifications need to be considered when making inferences about particular patterns that should be (or are) observed. First, I use a simplified representation of the networks themselves. Real regulatory networks are more dynamic than those considered here, increasing (or constraining) organism's ability to fine-tune given a particular environment at a particular time [47]. Furthermore, genes in real organisms are often co- or multiply-regulated: for example, the *even-skipped* gene that is part of the embryo patterning pathway in *Drosophila* development possesses at least seven enhancers that permit differential expression of the gene [48]. Together, these dynamics provide organisms phenotypic plasticity (developmental or otherwise) that allows them to cope with different selective challenges they face during their life (e.g., [49]). In this work I have assumed that each network confers an average phenotype during the individual's life that either meets or does not meet the average challenge presented by the environment. Future computational research should consider more realistic networks—combining the size of the networks considered here and the multiply-and feedback-regulated networks of previous researchers [27]–[29]—to analyze these different factors together and build towards a better set of theoretical expectations.

Second, I have removed all direct environmental effects on the GPM in order to focus on heritable variation. The indirect effect of selection removing particular genotypes from a parental generation is present, but environmental variance is an important aspect of heritability in nature. It seems likely that there is a point at which environmental variance will overwhelm any of the network-induced effects on heritability, but that remains to be explored. Keeping with the preceding paragraph, future research should consider these direct environmental effects.

Third, every gene in a Boolean network, in its raw form, is essentially equivalent with every other gene in the network: there are two alleles (0 and 1), and each is substitutable with one another in the network. In reality we know that a guanine nucleotide exchange factor won't become a heat shock protein as a result of a single mutation. As such, we have to recognize the real biochemical limitations that particular genes impose on the evolution of a quantitative trait, and recognize that there are regions of phenotypic space that may simply be inaccessible [50], [51]. That noted, all genes have evolved from some ancestral gene, and given sufficient time, a sequence may grow, shrink, or change nucleotide sequence to become another gene composed of the same four-letter alphabet. Unique molecular evolutionary histories resulting in phenotypic convergence are well-known [52], [53], but there are also several known cases of gene convergence from disparate taxa [54] and convergence in gene regulatory networks [55].

Finally, even though nature abhors a vacuum [56], in this paper I consider a single species existing in a single patch. Given that we know heritabilities for a quantitative trait can vary between species [7], and assuming that the genetic architecture of the traits plays a significant role in shaping heritability, we should ask why larger networks ever exist. According to this analysis, if a species' trait can adapt faster to a novel environment when the network is smaller, then all species should be driven to the smallest possible network for any trait in order to gain an adaptive advantage. But this is not the case. In forthcoming papers pending acceptance, I consider population dynamics in a fluctuating environment; competition between two species; and multiple-patch scenarios, the results of which refine the conclusions presented here.

In conclusion, I find: one, the size and topology of the network, plus the recombination rate in some cases, underlying a quantitative trait can strongly influence the trait's heritability. In particular, smaller, scale-free networks and low recombination rates increase heritability. In such networks, the rates of change of additive genetic variance and phenotypic variance are most dissimilar: networks allow phenotypic variance to change quickly while (hidden) additive variance changes slowly, thus resulting in higher heritability. Two, the effect of genetic architecture on heritability translates to altered population recovery times after sudden environmental changes, such that species with a small network tend to recover faster than species with larger networks. Conditional on further work refining these results, and in consideration of similar research focused on the density of connections in a network, these results provide a set of expectations bridging the genotype-ecotype map may be empirically tested.

## Materials and Methods

### Model Presentation

I focus on individuals of a single species living in a single patch with an environmental variable that remains constant through time (Hypothesis 1) or that changes from one steady state to a new steady state after a period of canalization (Hypothesis 2). The variable in the model is an environmental “driver”, that is, a variable whose value is not affected (or is minimally affected) by the presence or activity of individuals in the landscape. Examples of environmental drivers include temperature, salinity, and pH.

Individuals possess a single quantitative trait that maps to the environmental variable. For the three environmental driver examples above, this might include thermoregulatory ability, osmoregulatory ability, or ability to regulate pH. The trait is encoded by a directed Boolean network of 16, 32, 64, 128, or 256 genes, the state of each determined dynamically (see below). The topology of the network is initiated as either random (no preferential attachment) or scale-free (with preferential attachment) in its out-degree distribution [11]. Randomly-connected networks show an approximately Poisson degree distribution, whereas scale-free networks exhibit an power law degree distribution [57]. I use a lottery model algorithm to form the scale-free networks, i.e., the probability of an existing gene acquiring a connection to a new gene is proportional to the number of existing connections [57].

**Figure 7:**
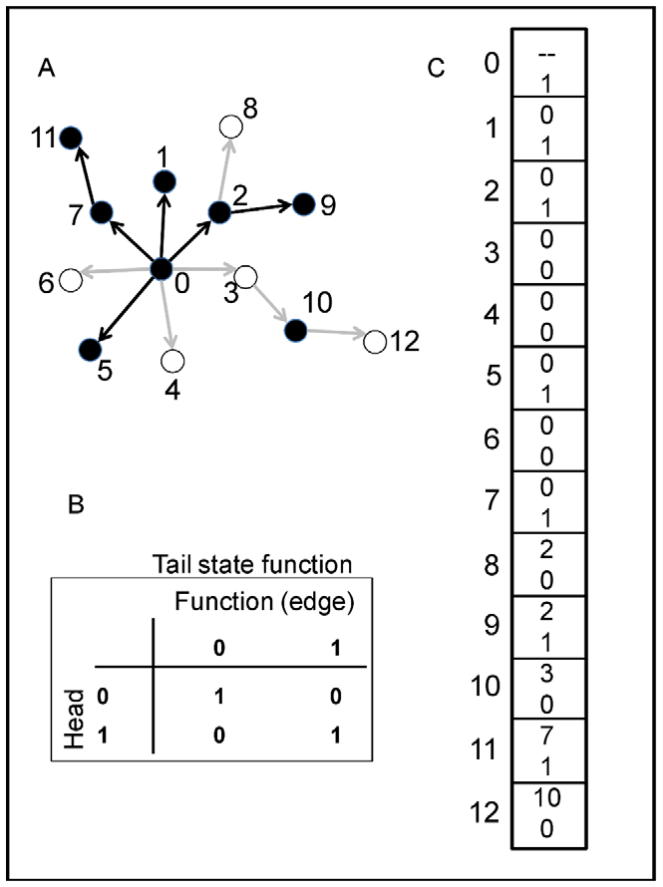
An example network, functional map, and chromosome. Part A shows an example 13-gene Boolean network. Black nodes are up-regulated (“on”; state = 1) genes and white nodes are down-regulated (“off”; state = 0). If an edge connecting two nodes is black, the “head” gene (upstream) activates the “tail” gene (downstream), and if an edge is gray, the head represses the tail gene. Part B provides the functional map; for example, if the head gene is “off” and the edge connecting the head and tail genes is an activator, then the tail gene is off (upper-right quadrant). Part C shows the chromosome corresponding to the network in Part A. Each block represents a gene (numbers along the left-hand side); within each block, the top number defines the “head” (i.e., immediately-upstream) gene while the bottom number defines the functional relationship (e.g., if 0, then the head gene is a repressor).

At the start of a run, every individual's network is randomly determined (as guided by the constraints of topological specification); with these relatively small populations, it is very unlikely that any two individuals possess the same exact network at simulation initiation. The binary state [0, 1] of each gene in the network except the upstream-most is determined by comparing the state of the gene immediately upstream to the functional relationship of the gene pair (Figure 7a, encoded by chromosome of 7c). The state of the upstream-most gene is determined randomly for each individual at simulation initiation, and is then inherited for subsequent generations. Some genes may act as repressors and others as activators, and the state of the downstream gene is determined by the match or mismatch between the state of the upstream gene and the function (Figure 7b). For example, if the upstream gene is “on” (state = 1) and is a repressor (function = 0), then the downstream gene takes the “off” state (state = 0). Alternatively, if the upstream gene state is 0 and it is a repressor, then the downstream gene takes the “on” state. Each gene except the basal-most has a single input to ease computational requirements (the number of calculations increases according to 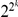 with *k* inputs [27]), but may have one or more outputs (i.e., may be pleiotropic). All network information is stored on a single chromosome consisting of two parts (Figure 7c). First, the topology is defined by a “tails list” of the downstream genes; the “heads list” (the controlling, upstream genes) is inferred from the index position of each tail list element. The relationship between heads and tails genes is randomly determined at the start of a simulation run, but, as noted above, the out-degree distribution is constrained by the scale-free versus random topological assignment. Figure 7a is an example 13-gene network whose states have been calculated given the information from the chromosome in Figure 7c.

Each individual's phenotype is determined by summing the states of all terminal genes in the network, i.e., genes with out-degree = 0, and scaling the value to the range of the environment ( = 140). So, for example, the network in Figure 7a possesses eight terminal genes, four of which are “on”, thus the individual possesses a phenotype of 70 ( = (140/8)*4). I am thereby assuming that there are no biochemical limits given a particular network size; individuals with a 16-gene network can approximate a phenotype of 140, as can individuals with a 256-gene network. The consequence for this re-scaling is that smaller networks have lower resolution than larger networks, which is a reasonable assumption given that dividing any particular task among fewer actors will result in lower overall accuracy. I stored the phenotypes of each individual's parents and used mid-parent regression to estimate the trait's heritability in the population. Additive genetic variance was derived by multiplying the phenotypic variance by the heritability.

Each individual's phenotype is translated to a fitness relative to the environment using a Gaussian function of the form,
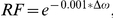
where Δ is the absolute value of the difference between the environment and the individual's phenotype, and ω is a value that changes the breadth of the selection function. I varied ω from 1.5 (high tolerance for a phenotype-environment mismatch) to 2.5 (low tolerance for a phenotype-environment mismatch) in the simulations. In this way I assume that the environmental effect is absolute and the phenotypic variance of the population plays no role in how an individual is selected. Each individual's *RF* does not affect the number of offspring produced, but does affect the probability that an individual will survive to reproduce.

Individuals are sexually-reproducing hermaphrodites who mate at random. The number of offspring from a mating is determined by drawing a random value from a Poisson distribution with λ = 1.5. Gametes undergo recombination during a diploid meiotic stage to create an offspring chromosome that is a mixture of parental alleles, which in this model are the tails list and the functional relationships. The first element of the offspring chromosome is chosen from the first element of one parent, then subsequent elements are taken from the same parent until a random uniform number less than the recombination rate (*r* = 0.05, 0.25, or 0.5) is drawn, at which point the element is drawn from the opposite parent. This continues the length of the chromosome. Mutation, as determined by testing a uniform random number against the mutation rate (1e^−3^, 1e^−4^, 1e^−5^) for each chromosomal element, occurs after the new chromosome is created. Although these mutation rates appear high, as noted by Frank [27], because the trait is directly related to fitness, the effective mutation rate is about one order of magnitude lower. All mutations are non-synonymous and may affect either the controlling function of a gene (an activator mutates to suppressor) or the relationship to another gene (i.e., alter network topology).

Death occurs after reproduction in three stages. First, all parents are killed to prevent over-lapping generations. Next, the new generation is culled according to each individual's relative fitness: if the *RF* is less than a uniform random number, then the individual dies. Last, a carrying-capacity is enforced by randomly killing individuals to bring the population below K = 500.

I examined patterns of epistasis in the gene networks by creating four identical individuals and randomly generating two mutations that would occur in either the functional relationship (activator versus repressor) or the topology of the network. I then mutated one individual with one mutation, mutated a second individual with the second mutation, and mutated the third individual with both mutations, creating both single mutants and a double mutant. The fitnesses of all four individuals was calculated as described above. Epistasis was measured as the fitness of the double-mutant minus the product of the single-mutant fitnesses, ε = *w_ab_*–*w_a_w_b_* [35]. I tested the role of network size (six levels), network topology (two levels), and the location of the mutations (functional relationship versus topological mutation) on epistasis using 1000 randomly-generated networks for each treatment, for a total of 24000 runs.

Treating the genetic architecture of a quantitative trait as a hierarchical network is inherently different than a purely linear (additive) system of the type central to classical analytical genetics. To test that the basic model structure (i.e., using a Boolean genetic system) did not violate the analytical results of Crow and Kimura [9] or Bürger [10]—specifically, that the rate of change of genetic variance was inversely related to the number of underlying loci—I changed the network model discussed above by defining the genetic architecture as a linear Boolean [0,1] string. The network representation of the GPM results in a mutational target size of 2*n*–1 because the edges between nodes can mutate (i.e., the relationship among the genes can change). To compensate for this near-doubling of mutational target size given network genetic architecture, I doubled the mutation rates from 0.001, 0.0001, and 0.00001 to 0.002, 0.0002, and 0.00002 in the linear genotype simulations. Recombination rates are unaffected by the difference because nodes and their relevant edges are coupled in the network model. Phenotypes were calculated as the sum of all loci scaled to the range of environmental values available, as in the network model; phenotypic variance was calculated directly from the population; heritability was calculated using mid-parent regression; and additive genetic variance was calculated as the product of phenotypic variance and heritability at each time step.

### Analysis

For all analyses, except where noted in the preceding text, the predictor variables were factors rather than continuous variables. Thus, although a nonlinear relationship is suggested in some figures, it was not required in the models. Although not presented, I also evaluated models in which network size was a continuous variable, and the inclusion of a second-order polynomial fit the data far better than a strictly linear model. For all analyses, I examined histograms, quartile-quartile plots, and predicted versus observed plots to ensure that model assumptions of normality (or Laplacian errors, in the cases of epistasis analysis [see below]) were met.

I tested the role of genetic architecture on epistasis using a generalized linear model (glm) with network size, network topology, mutation rate, and recombination rate as predictor variables. The epistasis data were highly kurtosed and exhibited extensive heteroskedasticity, with smaller networks showing higher variance in epistasis than larger networks. I used a Laplace distribution and an identity link function in the glm to accommodate the leptokurtosis. I weighted epistasis by its standard deviation within network sizes to homogenize variances.

I used two sets of simulations to test the effects of genetic architecture on rates of change of genetic and phenotypic variance, quantitative trait heritability, and population recovery times. In both sets, I initialized a population of 500 individuals in a single patch with the central environment value ( = 70) and ran the simulation for 250 generations. The value of the environment dropped suddenly either 20 or 30 units at generation 251 (i.e., after a 250-generation canalization period), and each simulation continued an additional 750 generations or until the population went extinct. For scenarios in which the genetic architecture was a network, the experimental design used five network sizes (*n* = 16, 32, 64, 128, 256); two topologies (scale-free or random); three mutation rates (1e^−3^, 1e^−4^, and 1e^−5^); two recombination rates (0.05 and 0.5); two fitness exponents (1.5 and 2); and two degrees of environmental change (20 and 30 units). I replicated each experiment three times, for a total of 720 simulations. I used the same experimental design as above in the scenario where genetic architecture was purely linear, except that there was no term for topology (total of 360 simulations). As discussed in Results, I extended the network simulations further by changing the environment when a particular amount of additive variance was reached by the population; analyses followed those described above.

I used only the pre-impact data (i.e., the first 250 generations) to estimate the effect of each predictor on rates of change of additive genetic and phenotypic variance, and quantitative trait heritability in a stable environment. I used a linear regression of additive genetic and phenotypic variance against time to estimate the rate of change for each run of the simulations. The rate of change of each parameter was then the response variable in a linear model. I used the average heritability over the last 100 generations as the response variable in a multi-way ANOVA to partition variation in heritability among the predictor variables. A slight skew in heritability distribution required log-transformation prior to analysis to ensure normally-distributed residuals.

I used the data from all time steps for each run to calculate recovery times. To set the baseline population to which the population had to return to be considered recovered, I first calculated the average population size for the 50 generations immediately preceding the environmental change. Time-to-recovery was then defined as the number of generations between the environmental change and the first generation in which the population had returned to the pre-change size. I used an ANOVA in which time-to-recovery was the response variable for both the network and linear network architectures. For some analyses, a full-interaction model resulted in far too many terms to be readily interpretable; I used Akaike's Information Criterion (AIC) to determine how different the best interpretable model was from the AIC-best model [58]. All simulations were run in NetLogo 4.1 [59], and all statistical analyses were completed in R 2.10 [60]. The epistasis analysis was completed using the VGAM package for R [61].
